# Acknowledgement to Reviewers of *Antibiotics* in 2013

**DOI:** 10.3390/antibiotics3010085

**Published:** 2014-02-27

**Authors:** Antibiotics Editorial Office

**Affiliations:** MDPI AG, Klybeckstrasse 64, CH-4057 Basel, Switzerland

The editors of *Antibiotics* would like to express their sincere gratitude to the following reviewers for assessing manuscripts in 2013:

Alfonzo, Antonio
Álvarez-Ordóñez, A.
Asuri, Prashanth
Bettina, Genthe
Björklund, Erland
Bohnert, Jürgen A.
Calokerinos, Anthony
Chachaty, Elisabeth
Chang, Feng-Yee
Charani, Esmita
Chen, Zhe-Sheng
Cheng, V. C. C.
Chou, Chi-chung
Cody, Vivian
Collins, James
Costelloe, Ceire
Courvalin, Patrice
Demirjian, Alicia
Doron, Shira
Dreier, Jürg
Drlica, Karl
Fatta-Kassinos, D.
Fralick, Joe A.
Gálvez, Antonio
Hall, Tony J.
Ho, Pak-Leung
Hughes, Diarmaid
Hung, Deborah T.
Huttner, Benedikt
Jungnickel, Christian
Kaatz, Glenn W.
Kiffer, Carlos
Kirst, Herbert A
Klitgaard, Janne
Koutchma, Tatiana
Krapac, Ivan G.
Leone, Marc
Lesprit, Philippe
Lin, Yu-Hsin
Llor, Carles
Lous, Jørgen
Mañes, Jordi
Martinez, Luis R.
Mazel, Diddier
Mcintosh, E. David G.
Moir, Donald T
Mueller, Eric W
Nielsen, Kaare M.
Nielsen-Preiss, Sheila M.
Oostenbrink, R
Opperman, Timothy J.
Pakhomova, Svetlana
Partridge, Sally R.
Perez, Astrid
Pérez, Lourdes
Pletz, Mathias
Pridmore, Andrew
Pruden, Amy
Qin, Zhiqiang
Rineh, Ardeshir
Rodriguez-Meizoso, Irene
Roemer, Terry
Roque, Fátima
Samaras, Vasilios
Samarasundera, Edgar
Sass, Peter
Schweizer, Herbert
Shigemura, Katsumi
Simpson, Jamie
Siqueira-Junior, José P.
Slain, Douglas
Solich, Petr
Solichova, Dagmar
Symmons, Martyn F.
Tatavarthy, Aparna
Theuretzbacher, Ursula
Thorsen, Line
Thulesius, Hans
Tiwari, Amit
Topp, Edward
Tsai, J.C.
Turner, R. Brigg
Van Der Velden, Alike
Victor, T. C.
White, Steve
Wood, Fiona
Woodward, Martin
Wu, Jiunn-Jong

